# Gender inequalities in occupational health related to the unequal distribution of working and employment conditions: a systematic review

**DOI:** 10.1186/1475-9276-12-57

**Published:** 2013-08-05

**Authors:** Javier Campos-Serna, Elena Ronda-Pérez, Lucia Artazcoz, Bente E Moen, Fernando G Benavides

**Affiliations:** 1 Center for Research in Occupational Health, Universitat Pompeu Fabra, Barcelona, Spain; 2 CIBER Epidemiología y Salud Pública (CIBERESP), Madrid, Spain; 3 Preventive Medicine and Public Health Area, University of Alicante, Alicante, Spain; 4 Agència de Salut Pública de Barcelona, Barcelona, Spain; 5 Institute of Biomedical Research (IIB-Sant Pau), Barcelona, Spain; 6 Research Group for Occupational and Environmental Medicine, Department of Public Health and Primary Health Care, University of Bergen, Bergen, Norway

**Keywords:** Gender identity, Occupational health, Socioeconomic factors, Work

## Abstract

**Introduction:**

Gender inequalities exist in work life, but little is known about their presence in relation to factors examined in occupation health settings. The aim of this study was to identify and summarize the working and employment conditions described as determinants of gender inequalities in occupational health in studies related to occupational health published between 1999 and 2010.

**Methods:**

A systematic literature review was undertaken of studies available in MEDLINE, EMBASE, Sociological Abstracts, LILACS, EconLit and CINAHL between 1999 and 2010. Epidemiologic studies were selected by applying a set of inclusion criteria to the title, abstract, and complete text. The quality of the studies was also assessed. Selected studies were qualitatively analysed, resulting in a compilation of all differences between women and men in the prevalence of exposure to working and employment conditions and work-related health problems as outcomes.

**Results:**

Most of the 30 studies included were conducted in Europe (n=19) and had a cross-sectional design (n=24). The most common topic analysed was related to the exposure to work-related psychosocial hazards (n=8). Employed women had more job insecurity, lower control, worse contractual working conditions and poorer self-perceived physical and mental health than men did. Conversely, employed men had a higher degree of physically demanding work, lower support, higher levels of effort-reward imbalance, higher job status, were more exposed to noise and worked longer hours than women did.

**Conclusions:**

This systematic review has identified a set of working and employment conditions as determinants of gender inequalities in occupational health from the occupational health literature. These results may be useful to policy makers seeking to reduce gender inequalities in occupational health, and to researchers wishing to analyse these determinants in greater depth.

## Introduction

The increase in women’s participation in the labour market has been one of the most important social phenomena of the second half of the twentieth century. For example, of the 3.0 billion people employed around the world in 2008, 1.2 billion were women (40.4%). That fact represents an increase of nearly 200 million women employed in the last 10 years. However, the gap in terms of activity, temporary employment and unemployment rates between women and men has remained stable worldwide [1]. For example, although women’s activity rate in the 27 European Union countries (EU-27) has increased by 2.6 per cent points from 2005 to 2011, the gap between men’s and women’s activity rates remained stable along this period, at around 12.6% in 2011. Moreover, of the total female working population in the EU-27 in 2011, 14.6% was working in temporary employments vs 13.6% of men. Also, the gender gap in the unemployment rate has traditionally been high in the EU-27, at around 1.4% points from 2005 to 2007. Nevertheless, this gender gap was reduced to 0.2% points in 2011 due to the economic global crisis, which has mainly affected the construction sector (a masculinized sector) in the EU-27 [2]. Furthermore, of the 550 million workers worldwide who are considered poor (workers who are unable to earn themselves and their families more than a 1 US dollar a day) 330 million (60%) are women [1].

One explanation for the origin of gender inequalities is structural, as the labour market has been organised on the pillars of a prevailing patriarchy and androcentrism. In addition, with the increasing insertion into the labour market of new groups of workers (among which women are the most important category) who have other attitudes about work and employment (departing from the lifelong, full-time career perspective), employers had an opportunity to develop a flexible employment regime. This latter regime, in turn, is also one of the sources of current inequalities in the labour market (i.e. between male and female workers) [3,4]. Beginning with the Industrial Revolution, a division of labour based on sex became the foundation on which gender inequalities were consolidated, confining women to domestic work and a family care-giver role (unpaid work) and men to paid work [5]. Women’s incorporation into paid work has not exempted them from unpaid work. They remain trapped in the family sphere, partly because they are bound by emotional ties to those for whose care they are responsible (“sticky floor”), as well as by an unequal distribution of domestic and family duties between partners [6]. Women and men entering the labour market also encounter feminised and masculinised sectors of activity (horizontal segregation), where women occupy the lowest positions on the decision-making scale (vertical segregation), and where professional promotion is hindered by invisible barriers of masculine power (“glass ceiling”) [7] and by language differences in speech styles between women and men (“wall of words”) [8]. All these conditions place women in a more precarious position than men [9,10]. For example, horizontal segregation produces a dense concentration of women in certain sectors of activity and in certain professions where the levels of remuneration are lower. Vertical segregation reinforces the effects of horizontal segregation, and also accounts for women’s lower wages [11,12]. In addition, women and men with the same job title usually perform different tasks, giving rise to an unequal distribution of working conditions and hazards between the two sexes, with a differential impact on their health [13,14]. For example, the job title of butcher is applied to women who work behind a delicatessen counter and interact with the public, and also to men who work behind a meat counter cutting large pieces of meat [15].

The consideration of a gender-based division of labour in occupational health studies not only implies separate analyses on the basis of sex, but must also take into account the potential different meanings of a given role for men and women in different social contexts, for example social classes and other dimensions of health inequalities. Moreover, research on gender inequalities in occupational health should tend to explain the complex pathways by which the social relations of gender may have an impact on the health of men and women workers. Therefore, consideration of the roles of both sex and gender is required [16,17].

In the last decade, several scientific studies in the field of occupational health have incorporated the gender perspective [18], although no study has been conducted to identify and collect all the work-related gender inequalities in health described in the literature. For example, several studies have indicated that employed women experience worse working conditions than men, and that a higher health burden might result from these exposures [19,20]. Determining the general picture of the existing work-related gender inequalities is of vital importance, not only for researchers and practitioners in occupational health, but also for policy makers, in order to optimise the efforts made by public administrations to reduce them.

Thus, the aim of this study was to identify and summarize the working and employment conditions described as determinants of gender inequalities in occupational health in studies related to occupational health published between 1999 and 2010.

## Methods

The study was based on a systematic review of observational studies. Ethics approval was not required for this study due to the fact that it is not an experimental study carried out on humans, while it is based in papers already published.

### Search strategy

**Figure 1:**
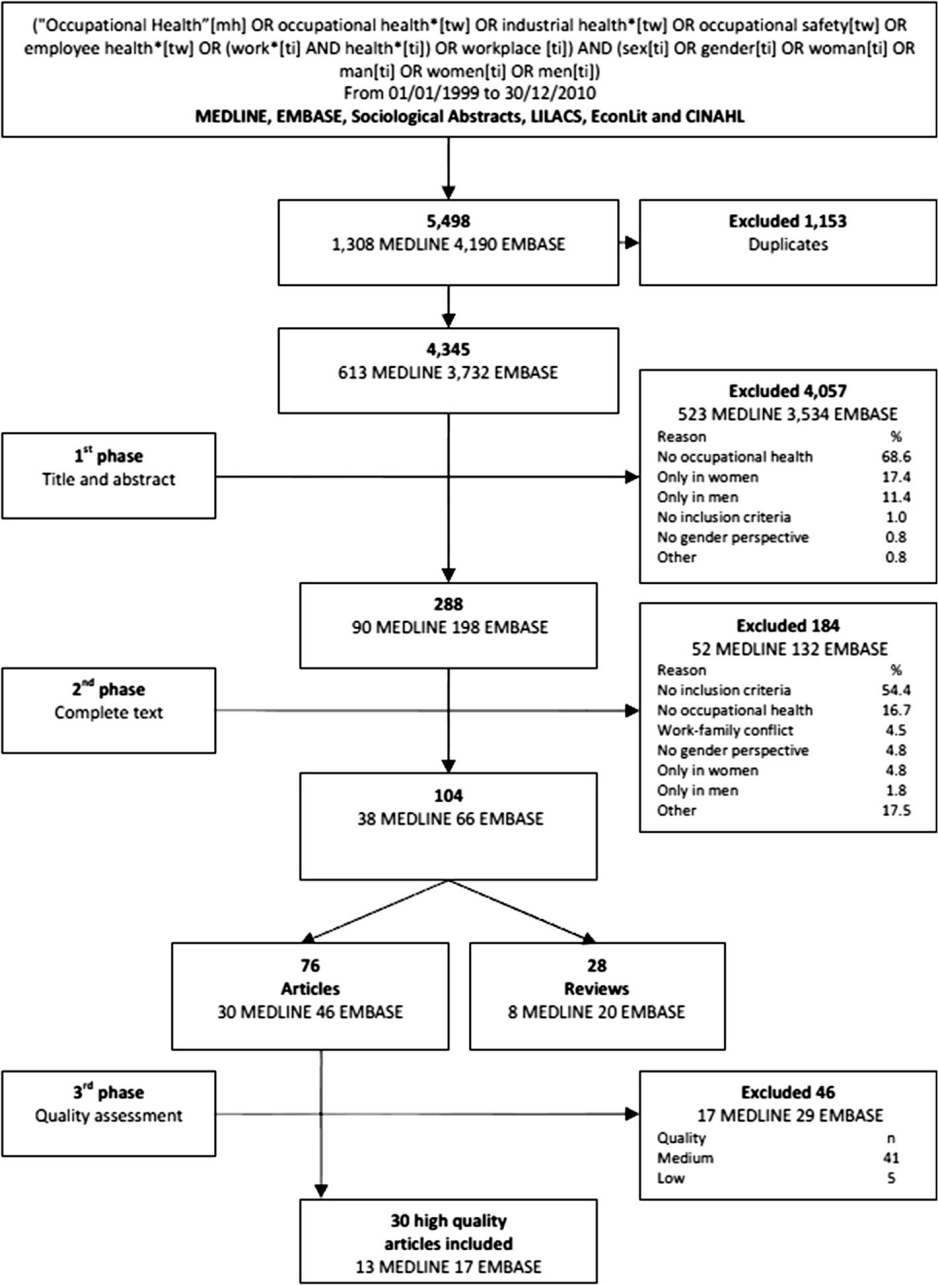
Flowchart followed in the selection process of the studies.

Electronic databases searched included MEDLINE (through PUBMED) EMBASE, Sociological Abstracts, LILACS, EconLit and CINAHL. The search was limited to publication dates from 01/01/1999 to 31/12/2010. This period was selected because it was during this time that the gender perspective was largely incorporated in the occupational health literature [18]. Keywords used were different terms reflecting gender and occupational health. The terms reflecting gender included: sex, gender, women, men, woman, man; the terms reflecting occupational health included: occupational health, industrial health, occupational safety, employee health, work, health, and workplace. The terms reflecting inequality (inequity, inequality and difference), were not used because otherwise they reduced significantly the results of the search conducted. The Boolean operators AND and OR were combined in a common search strategy in order to achieve the most sensitive, but not the most specific results in the search (Figure 1). Although the search strategy was not limited by the language of the publications, only articles in English or Spanish were selected, due to the researchers’ fluency in these languages.

### Studies selection process

This common search strategy identified 5,498 references, 1,308 in MEDLINE, 4,190 in EMBASE and none in the remaining databases (Figure 1). Of these, 1,153 duplicated articles were excluded. In the first phase, after applying the inclusion and exclusion criteria to the titles and abstracts (Table 1), the principal reviewer (JC) excluded 4,057 additional articles, for the most part because they did not focus on occupational health. In the second phase, after applying the selection criteria to the complete text of the remaining 288 articles, 104 were found to match the inclusion criteria. From these 104 articles, 28 were left out because they were reviews. Finally, a quality check was conducted during the second phase of the selection process. After going through the title and abstracts, 5% of the 288 included references were randomly selected. The two principal reviewers (JC and ER) independently applied the criteria for inclusion and exclusion throughout the complete text of the articles. The Kappa statistic for agreement between them was quite acceptable (k=0.85). All disagreements between JC and ER were due to different interpretations of one of the exclusion criteria, which were resolved after a short discussion. When this process was finished, the selection criteria were clarified and rewritten. Thus, it was not necessary to ask any other reviewer’s opinion since JC and ER reached agreement on all studies for which there was initial disagreement.

**Table 1:** Inclusion and exclusion criteria used in the selection process

**Inclusion criteria**	**Exclusion criteria**
Studies focusing on the differences between women and men in prevalence of exposure to occupational hazards related to working or employment conditions as determinants of health inequalities of working populations from a gender perspective	Studies not focusing on the differences between women and men in prevalence of exposure to occupational hazards related to working or employment conditions as determinants of health inequalities of working populations from a gender perspective
Studies in Spanish and English	Studies focusing only on women or men, but not both at the same time
	Studies including women and men, but without a gender perspective
	Study reviews
	Studies in occupational health focusing on specific biological differences between women and men such as pregnancy, birth, and breastfeeding
	Studies focusing on the unequal distribution between women and men of domestic and family tasks and their effects on health inequalities of working populations from a gender perspective

### Quality appraisal

In the third phase, the remaining 76 articles were critically and independently appraised by two reviewers (JC and ER) using two different specific standardised evaluation guidelines appropriate to the type of the epidemiological design of the study [21,22], both based on the STROBE statements [23].

The specific tool used to assess the quality of the 65 cross-sectional studiesl [21] comprised 27 items distributed in 8 domains with 6 categories of answer (poor, fair, good, very good, no information available, and not applicable). The domains were: a) *research question*, one item mainly evaluating whether the study is based on a clearly defined research question; b) *participants and internal validity*, five items mainly evaluating the sample adequacy and similarity to the base population and the control of selection bias; c) *comparability between groups*, four items mainly evaluating the study groups’ comparability and the control of selection bias; d) *study variables*, four items mainly evaluating the adequacy of the measurements of the main variables and the control of information bias; e) *statistical analysis and control of confounders*, four items mainly evaluating the adequacy of the analysis in measuring the control of confounding variables; f) *results*, four items mainly evaluating to which extent the results are well described, useful and precise; g) *conclusions*, four items mainly evaluating whether the results can be generalized to the population and to the context in which it aims to apply; and h) *conflict of interest*, one item evaluating whether the conflict of interests do not prejudice either the results or the conclusions of the study. The total quality score was determined as high-quality, if the majority (50% or over) of the 8 domains were classified as very good or good, unless the internal validity (evaluated through domains b to e) was classified as fair or poor; medium-quality, if the internal validity was classified as fair, or if the majority of the domains were classified as fair; and low-quality, if the internal validity was classified as poor, or if the majority of the domains were classified as poor. The internal validity was classified as fair or poor, when at least two of the four domains from b to e were scored as fair or poor, respectively.

The tool [22] used to assessed the quality of the five case–control studies included a checklist of 37 items distributed in 6 domains with 4 categories of answer evaluating if the domain was optimal or not (yes, no, partially and not applicable). The domains were: a) *research question* using three items; b) *methods and internal validity*, evaluating the participants with three items, selecting case and controls with 11, the groups’ comparability with two, the exposure with four, and the statistical analysis with five; c) *results* using five items; d) *conclusions*, using one; e) *conflict of interest*, using two; and f) *external validity*, using one.

The tool [22] to assess the quality of the six cohort studies used a checklist of 49 items distributed in 6 domains with 4 categories of answers evaluating if the domain was optimal or not (yes, no, partially and not applicable). The domains were: a) *research question*, using four items; b) *methods and internal validity*, mainly evaluating the participants with six items, the groups’ comparability with three, the exposure with nine, the effects with four, the groups’ monitoring with seven, and the statistical analysis with six; c) *results*, using six items; d) *conclusions* using one; e) *conflict of interest* using two; and f) external validity using one.

In both case–control and cohort studies, all six domains were taken equally into account to classify them as high, medium or low-quality studies. They were classified as high-quality, when five or more of any of the six domains were assessed as optimal; medium-quality, when three or four domains were assessed as optimal; and low-quality, when only one or two domains were assessed as optimal.

After this assessment, 41 medium and 5 low-quality studies were rejected, because the review team decided to limit the focus to articles with the highest standards of quality. Twenty-nine high-quality studies were finally included in our review.

### Identifying working and employment conditions as determinants of gender inequalities

We obtained a set of descriptive variables to characterize each of the 30 studies included in the review: first author, year of publication, country where the study was conducted, study design, study population, main dimension assessed, sample size, main objective, main findings and the relevance for the review. We also calculated the frequency of the dimension and subject mainly examined. Finally, we conducted a qualitative analysis of the descriptive results of each of the 30 studies. We focused on the differences observed between women and men in the prevalence of exposure to different working and employment conditions as determinants of gender inequalities in occupational health. In addition, we focused on the differences between women and men in the prevalence of work-related health problems as outcomes in each of the 30 studies. The statistical significance of each of the differences observed in the prevalence (p-value) was considered in the results and discussion section of this manuscript, but not in the selection process of the differences observed in the studies. Gender differences in the prevalence of exposure to the working and employment conditions were selected when the same gender difference appeared in two or more of the 30 studies included in the review, regardless of whether the difference in prevalence was statistically significant. We considered that a difference between women and men in the prevalence of exposure to the working and employment condition or in the work-related health problems was an inequality when it was avoidable, unfair, and systematically affected a higher proportion of women than men or vice-versa [24]. We grouped gender inequalities identified in the three dimensions analysed in this review: working conditions and employment conditions as determinants of gender inequalities in occupational health and inequalities related to work-related health problems as outcomes; including physical and mental health.

## Results

### Studies characteristics

Most of the 30 studies included in the review [25-54] were conducted in Europe (n = 19) and the United States (n = 5). The most frequent design was cross-sectional (n = 24), followed by cohort (n = 4) and case–control (n = 2). Most of the studies (73.3%) used samples of more than 1,000 people and were based on some type of working population (Table 2).

**Table 2:** Characteristics of the 30 studies included in the systematic review

**First author**, **year**	**Country**	**Design**	**Study population**	**Dimension assessed**	**Sample**
Gadinger 2010, [25]	Germany	Cross-sectional	middle / top managers	Working conditions	424
Persson 2009, [26]	Denmark	Cross-sectional	Manufacturing of rubber and mechanical assembly	Working conditions	33
Taiwo 2009, [27]	United States	Cohort	Aluminium employees	Working conditions	9,527
Hooftman 2009, [28]	Netherlands	Cohort	Any	Working conditions	1,578
Hooftman 2009, [29]	Netherlands	Cross-sectional	Any	Working conditions	80
Galanakis 2009, [30]	Greece	Cross-sectional	Any	Working conditions	2,775
Alterman 2008, [31]	United States	Cross-sectional	Farm operators	Work-related health problems	7,137
Kim 2008, [32]	South Korea	Cross-sectional	Any	Employment conditions	2,608
Lin 2008, [33]	Taiwan	Cross-sectional	Any	Work-related health problems	1,890
Magnusson 2008, [34]	Sweden	Cohort	Any	Working conditions	3,004
Artazcoz 2007, [35]	Spain	Cross-sectional	Salaried contract workers	Working conditions	2,792
Li 2006, [36]	China	Cross-sectional	Physicians	Working conditions	522
Peter 2006, [37]	Sweden	Case control	Any	Working conditions	1,381
Aittomaki 2005, [38]	Finland	Cross-sectional	Employees between 40–60 year old	Working conditions	5,802
Leijon 2005, [39]	Sweden	Cross-sectional	Any	Working conditions	156
Ludermir 2005, [40]	Brazil	Cross-sectional	Any	Employment conditions	683
Artazcoz 2005, [41]	Spain	Cross-sectional	Salaried workers	Employment conditions	2,472
Borrell 2004, [42]	Spain	Cross-sectional	Any	Working conditions	4,219
Lallukka 2004, [43]	Finland	Cross-sectional	Any employed person between 40–60 years old	Working conditions	6,243
Melamed 2004, [44]	Israel	Cross-sectional	Industrial sector workers	Working conditions	5,727
O’Campo 2004, [45]	United States	Cross-sectional	Any	Employment conditions	1,105
Cortès 2004, [46]	Spain	Cross-sectional	Any	Employment conditions	4,158
Muhonen 2003, [47]	Sweden	Cross-sectional	Workers in the sales division of a telecom company	Working conditions	279
Bildt 2002, [48]	Sweden	Cohort	Any	Working conditions	420
Karlqvist 2002, [49]	Sweden	Cross-sectional	Visual device units operators	Working conditions	1,283
de Zwart 2001, [50]	Netherlands	Cross-sectional	Any	Work-related health problems	16,874
Ibrahim 2001, [51]	Canada	Cross-sectional	Any	Working conditions	8,273
Islam 2001, [52]	United States	Cross-sectional	Any	Work-related health problems	40,193
Dosemeci 1999, [53]	United States	Case control	Any	Working conditions	1,125
Emslie 1999, [54]	United Kingdom	Cross-sectional	University workers	Work-related health problems	1,641

Table 3 shows main objective, findings, relevancy and quality score of each study included in the review. Most of the studies (n = 24) were focused mainly on how differences between women and men in the exposure to any kind of occupational hazards impact on their physical and mental health; another four [26,27,29,54] out of the 30 studies examined whether women and men with similar work tasks exhibit differences in their health impact; finally, only two studies [33,52] looked at injuries related to occupational accidents. Only one study [42] introduced the occupational social class as the main factor explaining gender differences in the exposure to working conditions and their impact on health. The quality score of most of the studies (n = 28) was over 83%.

**Table 3:** Description of the 30 studies included in the systematic review

**First author**, **year**	**Main objective**	**Main findings**	**Relevancy for the review**	**Quality score**
Gadinger 2010, [25]	To investigate cross-sectional associations between main, interactive and gender-dependent effects of the demand–control–support (DCS) model and subjective health in managers	Job demands appear to have a higher impact on psychosomatic complaints than job control and social support. No significant main effect of gender was observed in the prediction of psychosomatic complaints and self-rated health. High social support and male gender were found to buffer the increasing prevalence of psychosomatic complaints resulting from high work demands. In contrast, no significant two-way interaction was found in the prediction of self-rated health	This study analyses the differences between female and male managers in exposure to job strain and how it impacts differently on their health. It concluded that high job control and high social support may buffer adverse health effects that are associated with demanding jobs and that special attention should be given to isolation in women	6/8
Persson 2009, [26]	To examine whether men and women with the same job tasks exhibit differential physiological and psychological activation to manual and repetitive labour	Men and women respond to the work situation in a similar way. Only with regard to reports of positively valued high activity states, did men and women show a differential response. Accordingly, while men reported lower energy scores at the end of the work shift, women showed only a slight decrease	The interest of this study lays in the fact that it analyses the differential effect that exposure to the same job tasks has on women and men, not only psychological, but also physiological activation	8/8
Taiwo 2009, [27]	To determine if female workers in a heavy manufacturing environment have a higher risk of injury compared with males when performing the same job and to evaluate sex differences in type or severity of injury	Female workers in this industry have a greater risk for sustaining all forms of injury than male. This excess risk for female workers persisted when injuries were dichotomized into acute injuries and musculoskeletal disorder related injuries	This study provides evidence of a sex disparity in occupational injury with female workers at higher risk compared with their male counterparts in a heavy manufacturing environment	6/6
Hooftman 2009, [28]	To determine whether there are gender differences in the effect of exposure to work-related physical and psychosocial risk factors on low back, neck, shoulder, or hand-arm symptoms and related sickness absence	Except for the effect of bending the wrist and the neck backwards, men generally have a higher risk of symptoms with equal exposure	Although women are expected to be more vulnerable to exposure to work-related risk factors, the results of this study showed that, in many cases, men are more vulnerable. Thus, this study could not explain gender differences in musculoskeletal symptoms among workers	5/6
Hooftman 2009, [29]	To determine whether men and woman with equal tasks perform these tasks in the same way	When level, duration and frequency of exposure were analyzed at the same time, men and women had slightly different exposure patterns. However, these differences were not found when duration and frequency were analyzed separately.	This study conclude that gender differences in the exposure to ergonomics hazards within the same task cannot alone explain gender differences in musculoskeletal symptoms	7/8
Galanakis 2009, [30]	To examine gender differences in occupational stress, taking into consideration the role of marital status, age and education	Women appear to experience significantly higher levels of occupational stress. But when age, marital status and educational level are controlled for, there is no significant gender difference in occupational stress	This study shows that gender differences in stress do not stem from a genetic or biological difference. Gender differences in stress seem to reflect social and psychological differences associated with age, marital status and education. As environmental demands outside the family have pronounced effects related to stress in the family, the opposite is also true. Stress experienced in the family crosses over to the workplace	7/8
Alterman 2008, [31]	To collect baseline prevalence data on the work-related health problems faced by minority, white and female farm operators	Men and women of the same race or ethnicity showed statistically significant differences in the prevalence of many health conditions. Women reported more respiratory symptoms and musculoskeletal diseases in contrast to men, who had greater impairment of hearing acuity	The article focuses on how women and men of the same race/ethnic group present different work-related health problems	5/8
Kim 2008, [32]	To examine whether nonstandard workers reported poorer health compared to standard workers and assess whether there are gender differences in the association between employment status and chronic health outcomes	Male nonstandard workers exhibited a strong association with musculoskeletal disorders and liver disease, while women showed an association between nonstandard work and mental health disorders	The article focuses on how poor working employment conditions affects differently women and men’s health	8/8
Lin 2008, [33]	To provide an epidemiological basis for gender-specific work-related accident prevention programs	The male fatality rate from occupational accidents is almost eight times higher than in females. Females injuries were more common in such industries as construction, manufacturing and services, while male injuries were more common only in construction and manufacturing, but not in services	This study adds the gender perspective to the analysis of work-related accidents. It compare male and female occupational deaths and injuries and the type of industry where the accident occurs	7/8
Magnusson 2008, [34]	To investigate the association between demand, control, support and conflicts, downsizing and emotional exhaustion in men and women in a representative sample of the working population in Sweden	Work-related psychosocial hazards are prospectively associated with emotional exhaustion, but with differences between women and men. For men, lack of support from superiors seemed more predictive of exhaustion, while the opposite tendency was seen for women	The interest in this study is based in the fact that it analyses the different distribution of work-related psychosocial hazards between women and men and its differential impact on their mental health	6/6
Artazcoz 2007, [35]	To analyze gender differences in the impact of long workhours on a variety of health outcomes and health-related behaviour in salaried workers in Catalonia	Health factors associated with long workhours differed by gender. Whereas among the men, long workhours were only associated with a shortage of sleep, among the women they were related in addition to: poor mental health, hypertension, job dissatisfaction, smoking and lack of leisure-time physical activity. This consistent pattern among the women was only partially accounted for by domestic work	This study focuses on how long workhours are differently distributed between women and men and the differential impact on women and men’s health and health behaviour. Furthermore, it highlights the importance of also analysing domestic environment in these kinds of studies	8/8
Li 2006, [36]	To analyse the association between work stress, measured by job strain and effort-reward imbalance, and health in a sample of hospital-based Chinese physicians	Job strain and effort-reward imbalance were associated with impaired health functioning in women and men, but effort-reward imbalance showed a stronger association. Men’s job control was pronouncedly higher, and was related to men’s physical health; whereas women perceived relatively higher reward, which predicted women’s mental health	The interest of this study is based on the fact that it analyses the different distribution of work-related psychosocial hazards between women and men and its differential impact on their mental and physical health	8/8
Peter 2006, [37]	To investigate whether occupational gender segregation moderates the association between job stress in terms of effort-reward imbalance and the risk of myocardial infarction	The strongest association between myocardial infarction and overcommitment was found among women working in male-dominated jobs. Moreover, a significant multiplicative interaction of overcommitment and male domination in relation to myocardial infarction was observed in women	This study analyses the different distribution of work-related psychosocial hazards between women and men and its differential impact on myocardial infarction and how it is modulated by male and female-dominated jobs	6/6
Aittomaki 2005, [38]	To test whether higher age is associated with a lower prevalence of physically demanding work; and whether physically demanding work is more strongly associated with limited functioning in older employees than their young counterparts from a gender perspective	Among women, physical workload was more strongly associated with limitations in daily activities among older than younger employees. However, among men, the opposite was observed. It is possible that fewer men than women are still employed in physically demanding occupations at high age. Physical workload and possibilities to adapt to lower work capacity among older employees probably involve gender differences that are so far unknown	The study introduces the gender perspective in the exposure to physical demanding work in older workers	8/8
Leijon 2005, [39]	To investigate if and how exposure to sitting/standing, awkward arm and trunk postures and movements are associated with occupational gender segregation	The association between exposure and occupational gender segregation was strongest within female-dominated jobs. Workers with a low status/ authority in these jobs had the highest overall exposure levels	The study analyses differences between women and men in the exposure to awkward work postures and occupational gender horizontal and vertical segregation	8/8
Ludermir 2005, [40]	To investigates the gender difference in the association between employment status and common mental disorders	The relationship between unemployment and common mental disorders was stronger among females than among males. Additionally, the association between informal work and common mental disorders appears to be absent in males, while it was high for females	This study is one of the few that provides some evidence of a gender difference in the association between informal work and common mental disorders	8/8
Artazcoz 2005, [41]	To analyze the impact of flexible employment on mental health and job dissatisfaction; and to examine the constraints imposed by flexible employment on men’s and women’s partnership formation and people’s decision to become parents. For the two objectives the potentially different patterns by sex and social class are explored	Whereas non-fixed term contracts and working with no contract were associated with poor mental health status, no association with fixed term temporary contracts was seen. The effect of flexible contractual arrangements, other than fixed term temporary contracts, on mental health was higher among less privileged groups (women and manual male workers) and the impact of flexible employment, either fixed term or non-fixed term contracts, in family formation was more pronounced among men	This study is one of the few which examine the impact of flexible employment on workers’ health and wellbeing in Spain, the country with the highest rate of temporary contracts in the EU-15. In contrast with many studies based on self perception of job instability, this study focuses on an objective indicator, type of contract	8/8
Borrell 2004, [42]	To analyse the association between self-reported health status and social class and to examine the role of work organization, material standards and household labour as potential mediating factors in explaining this association from a gender perspective	Among men, work organisation seems to be an important mechanism that translates higher working class positions into better health. Among women, the association between poor health and working class position seems to be accounted for not only by hazardous forms of work organisation but also by household characteristics, household material standards and excessive amounts of uncompensated household work	In this study household labour and household standards of living have been included together with work organisation as possible mediating mechanisms of the relation between social class and health of the working population	8/8
Lallukka 2004, [43]	To analyse whether unfavourable working conditions are associated with diet, physical activity, alcohol consumption and smoking	Job strain was associated with all the studied health behaviours among women, but not among men. Low job strain was associated with healthy diet, high physical activity and nonsmoking	This study analyses the different distribution of exposure to work-related psychosocial and physical hazards between women and men, and how this different distribution in the exposure influences their health behaviours	8/8
Melamed 2004, [44]	To explore the possibility that exposure to noise at work might interact with job complexity and gender to affect the incidence of occupational injury among industrial employees	In high noise and high job complexity women showed higher risk from injury relative to those women in the less noise and less job complexity. The corresponding risk in men in high noise and high job complexity was less than half	This study analyses from a gender perspective the differences in the exposure to an environmental risk factor in occupational health to which men have traditionally had higher exposure than women	8/8
O’Campo 2004, [45]	To explore the conceptualization and measurement of gender inequality in the workplace and how these inequalities may impact health by the creation of indicators of gender inequality in the workplace	Wide gender inequalities between women and men within occupational categories were found in terms of pay, position of power, supervisory responsibilities, jobs with high strain and jobs that are passive. In general, women are more likely to have passive jobs, to receive lower pay, to occupy jobs with fewer policy-making responsibilities and jobs with fewer supervisory responsibilities	This is one of the first studies to describe gender inequalities in terms of pay, power and job stress within occupational categories for the purpose of examining associations with women’s health status	7/8
Cortès 2004, [46]	To analyze inequalities in mental health in the working population by gender and professional qualifications, and to identify psychosocial risk factors and employment conditions related to the mental health of this population	Women were more likely to report poor mental health status than men, although sex differences were greater among manual workers. Differences according to qualifications were found among women only in those working in manual jobs compared to women working in non-manual jobs, while no differences were found among men according to qualifications. Mental health is worse in women, and a relationship with professional qualifications is observed only in this group; women with less skilled occupations have poorer mental health status	This study is one of the few that analyses the association of working and employment conditions and mental health from a gender perspective while taking into account occupational social class	7/8
Muhonen 2003, [47]	To investigate the main and the interaction effects of the demand-control-support model on women’s and men’s health in a Swedish telecom company	Demands had main effect for women’s health symptoms, whereas both demands and lack of social support acted as predictors for men’s health symptoms. Control did not predict health either for women or men	This study focuses mainly in the differences between women and men in exposure to the demand-control-support model and how this impacts women’s and men’s health	8/8
Bildt 2002, [48]	To examine how working conditions in 1993 influenced the occurrence of poor mental health in 1997 among women and men	Shift work, job strain, no education at the employer’s expense, low occupational pride, low stimulation at work and poor social support were related to poor mental health among women, while among men, only shift work and low occupational pride were found as risk indicators of poor mental health	Interesting study focusing on how the different distribution of employment and working conditions impacts on women and men’s mental health	7/8
Karlqvist 2002, [49]	To describe working conditions and the prevalence of musculoskeletal symptoms among male and female visual device units operators, and to assess associations between work-related physical and psychosocial exposures, respectively, and neck and upper limb symptoms, and whether these associations differed between women and men	More women compared to men were exposed to organizational, physical and psychosocial working conditions that have been recognised as harmful conditions in previous studies published in occupational health scientific research	This study analyses the different distribution of exposure to work-related physical and psychosocial hazards between women and men; and how it impacts differently women’s and men’s physical health	8/8
de Zwart 2001, [50]	To analyse the association between gender and upper extremity musculoskeletal complaints, among the general working population and in various occupational groups. To test whether the higher risk in women in the general working population for these types of complaints can be explained partly by differences in the distribution of male and female workers in occupations with different risks for the onset of upper extremity musculoskeletal complaints	Female workers showed a consistently higher risk of complaints of the upper extremities among the general working population as well as in many occupational social classes. Gender differences in musculoskeletal disorders are independent of the type of occupation. On the other hand, the theory of gender segregation in work tasks among employees in the same job title may also still be a plausible explanation for our findings	This study confirmed the presence of gender differences in upper extremity musculoskeletal complaints among working populations as well as within several occupational classes, with women reporting a higher number of symptoms. The results, however, do not lend support to the hypothesis that women suffer more musculoskeletal complaints due to gender segregation of the labour market, which places women in occupations with higher risk of being exposed to musculoskeletal hazards. Potentially, it can be attributed to differences in work-related and non-work-related factors between sexes	7/8
Ibrahim 2001, [51]	To explore the association, for working women and men, of high strain jobs with self-rated health in the 1994/1995 Canadian National Population Health Survey	The job strain and poorer self-rated health relationship was consistent across both levels of poorer health. The relationship was weaker for women than men despite the fact women reported higher psychosocial demands and lower control than men	This study mainly analyses how differences in the exposure of women and men to job strain and job insecurity impacts on their self-perceived health	7/8
Islam 2001, [52]	To examine the overall work-related injuries in an exclusively state-funded workers compensation system that allows comparison of compare injuries/illness rates, types, causes, and effects in major occupations and industries between males and females	Among the compensable injury/illness cases, a greater proportion of females than males had back, ankle, hand, neck, shoulder and wrist injuries. The incidence of musculoskeletal disorders was significantly higher in females than males working in the service industry	This study analyses the different distribution of work-related injuries and illnesses between women and men	7/8
Dosemeci 1999, [53]	To analyse gender differences in the risk of renal cell carcinoma and occupational exposures to chlorinated aliphatic hydrocarbons	More men than women were exposed to organic solvents in general. However, the risk of renal cell carcinoma was significantly elevated among women exposed to all organic solvents combined. Among men exposed to any of the organic solvents, no significant excess risk was observed	This is one of the first studies to introduce the gender perspective in analysing how differences between women and men in the exposure to a chemical hazard can explain its different impact on a specific occupational illness	5/6
Emslie 1999, [54]	To examine gender differences in minor morbidity among men and women working in similar circumstances, and to test whether the relation between reported working conditions and health is similar for men and women	Female university employees reported more physical symptoms and more malaise symptoms than male employees, but mean scores on measures of minor psychiatric morbidity did not differ by gender	This study concludes that the gender differences observed in health complaints is due to gender differences in the exposure to worse working conditions, rather than to a differential vulnerability between sexes.	8/8

In general terms, the studies included in the review covered a wide range of dimensions and subjects (Table 4). Most were related to the dimension of working conditions as a determinant of gender inequalities in occupational health (n = 20). Of these 20 studies, eight [25,30,34,36,37,47,48,51] focused mainly on the differences between women and men in the exposure to work-related psychosocial hazards and how that fact impacts on their health. Five [25,34,47,48,51] of these 20 studies were based on the demand-control-support model, two of which followed a cohort design [34,48]. Another two studies [36,37] of these 20 were based on the effort-reward imbalance model, one of which followed a case–control design [37]. A smaller number of studies [32,40,41,45,46] (n = 5) were related to the dimensions of employment conditions as a determinant of gender inequalities in occupational health and another 5 studies [31,33,50,52,54] examined how work-related health problems are differentially reported by employed women and men. Only one of the 30 studies included in the review focused on social class position [42], time spent in paid work [35] and work organization [45] as the main explanatory variables for the gender inequalities observed.

**Table 4:** Frequency of the subject mainly examined in the 30 studies included in the systematic review

**Dimension and subjects**	**Study**^**a**^	**N**^**b**^
**Working conditions**		**20**
• Psychosocial hazards		
• Demand-Control-Support	Gadinger [25], Magnusson [34]^c^, Muhonen [47], Bildt [48]^c^, Ibrahim [51], Galanakis [30]	
• Effort-Reward Imbalance	Li [36], Peter [37]^d^	
• Physical hazards	Persson [26], Hooftman [29], Aittomaki [38], Leijon [39]	
• Psychosocial/physical hazards	Hooftman [28]^c^, Lallukka [43], Karlqvist [49]	
• Environmental hazards	Taiwo [27]^c^, Melamed [44], Dosemeci [53]^d^	
• Social class position	Borrell [42]	
• Time spent in paid work	Artazcoz [35]	
**Employment conditions**		**5**
• Employment status	Kim [32], Ludermir [40], Artazcoz [41], Cortès [46]	
• Work organization	O’Campo [45]	
**Work**-**related health problems**		**5**
• General health complaints	Alterman [31], de Zwart [50], Emslie [54]	
• Injuries or illnesses	Lin [33], Islam [52]	
Total		**30**

a Study included in the review with the first author and the reference number.

b Number of studies.

c Cohort design; d Case–control design.

Table 5 shows gender differences in the prevalence of exposure to a series of occupational hazards related to working and employment conditions and differences between women in men in reporting work-related health problems. Differences between women and men in the exposure to the demand-control-support model were analysed in 12 out of the 30 studies included in the review. In addition, differences in reporting musculoskeletal symptoms were analysed in 7 out of the 30 studies. More men than women were exposed to low support in two studies with a cohort design [34,48], high physically demanding work in a study with a cohort design [48] and effort-reward imbalance in a study with a case–control design [37]. Conversely, more women than men were found to work with a temporary contract and in a shift-work in a study with a cohort design [48]. In addition, more women than men were found to report musculoskeletal symptoms in a study with a cohort design [28].

**Table 5:** **Frequency and percentage of the 30 studies included in the review which showed gender differences in the prevalence of exposure to occupational hazards related to working and employment conditions and work**-**related health problems**

**Occupational hazards and work**-**related health problems**^**a**^			**Studies which showed gender differences in the prevalence**	
	**n**^**b**^	**%**^**c**^	**Higher prevalence of women than men**	**Higher prevalence of men than women**
**Working conditions**				
*Demand*-*Control*-*Support*	12	40.0		
High demand and low control			Bildt [48]^Φd^; Karlqvist [49]^Φ^; Ibrahim [51]^Φ^	Lallukka [43]^Φ^; O’Campo [45]^Φ^
High demand			Gadinger [25]^**^; Magnusson [34]^Φd^; Karlqvist [49]^Φ^	Li [36]^ψ^ Borrell [42]^Φ^ Cortès [46]^Φ^ Muhonen [47]^*^
Low control			Magnusson [34]^Φd^ Li [36]^***^ Borrell [42]^Φ^ Cortès [46]^Φ^ Muhonen [47]^*^ Karlqvist [49]^*^	Gadinger [25]^*^
Low support				Gadinger [25]^*;^ Magnusson [34]^Φd;^ Cortès [46]^Φ;^ Muhonen [47]^*^; Bildt [48]^Φd^; Karlqvist [49]^Φ^
*Time spent in paid work*	5	16.7		
Long workhours				Artazcoz [35]^***^; Li [36]^**^; Lallukka [43]^Φ^; Bildt [48]^Φd^; Karlqvist [49]^Φ^
*Physically demanding work*	3	10.0		
High physically demanding work				Lallukka [43]^Φ^; Bildt [48]^Φd^; Ibrahim [51]^Φ^
*Noise at work*	3	10.0		
High noise at work				Borrell [42]^Φ^; Melamed [44]^**^; Karlqvist [49]^Φ^
*Job insecurity*	2	6.7		
High job insecurity			Borrell [42]^Φ^; Ibrahim [51]^Φ^	
*Effort*-*Reward imbalance*	2	6.7		
Effort-reward imbalance				Li [36]^*^; Peter [37]^*e^
**Employment conditions**				
*Type of contract*	5	16.7		
Permanent				Kim [32]^Φ^; Artazcoz [41]^Φ^
Temporary			Kim [32]^Φ^; Borrell [42]^Φ^; Cortès [46]^Φ^; Bildt [48]^Φd^	
Temporary fixed term			Artazcoz [41]^Φ^	
Temporary non-fixed term				Artazcoz [41]^Φ^
No contract			Artazcoz [41]^Φ^; Cortès [46]^Φ^	
*Job status*	4	13.3		
Supervisors				O’Campo [45]^Φ^; Ibrahim [51]^Φ^
Upper manager				Gadinger [25]^**^; Muhonen [47]^*^
Middle manager			Ibrahim [51]^Φ^	
*Employment status*	3	10.0		
Full-time				Kim [32]^Φ^; Cortès [46]^*^
Part-time			Kim [32]^Φ^; Cortès [46]^Φ^; Ibrahim [51]^Φ^	
*Shift work*	2	6.7		
Shift-work			Bildt [48]^Φd^	Cortès [46]^Φ^
**Work**-**related health problems**				
**Physical health**				
*Musculoskeletal symptoms*	7	23.3		
Any			Alterman [31]^*^; Kim [32]^Φ^; Muhonen [47]^ψ^; Karlqvist [49]^*^; Emslie [54]^**^	
Low back			Hooftman [28]^**d^; Karlqvist [49]^*^	
Neck			Hooftman [28]^**d^; Karlqvist [49]^*^; de Zwart [50]^**^	
Upper extremity				
Shoulder			Hooftman [28]^**d^; Karlqvist [49]^*^; de Zwart [50]^**^	
Arm-hand			Hooftman [28]^**d^; Karlqvist [49]^*^	
Wrist			de Zwart [50]^**^	
*Self*-*perceived physical health*	3	10.0		
Self-perceived poor health			Gadinger [25]^**^; Kim [32]^Φ^; Ibrahim [51]^Φ^	
*Respiratory symptoms*	2	6.7		
Respiratory symptoms			Alterman [31]^*^	Kim [32]^Φ^
**Mental health**				
*Self*-*perceived mental health*	5	16.7		
Poor mental health status			Artazcoz [41]^Φ^; Cortès [46]^Φ^; Emslie [54]^ψ^	
Mental health disorder			Kim [32]^Φ^; Ludemir [40]^*^	
*Psychosomatic complaints*	2	6.7		
Psychosomatic complaints			Gadinger [25]^**^; Emslie [54]^**^	
*Self*-*reported occupational stress*	2	6.7		
Self-reported occupational stress			Galanakis [30]^**^; Ibrahim [51]^*^	

a To be included, the difference between women and men in the prevalence of the exposure to the occupational hazard or the work-related health problem must be shown in at least two studies; b Number of articles where the difference in the prevalence was shown; c Percentage of articles where the difference in the prevalence was shown; d Cohort studies; e Case–control design.

* p < 0.01; ** p < 0.001; *** p < 0.0001; ^Ψ^ p ≥ 0.05; ^Φ^ No information available on the p-value or any other parameter of statistical significance regarding the difference observed in the prevalence.

### Working conditions and gender inequalities in occupational health

Whereas the studies that analyzed the differences between women and men in the prevalence of exposure to work-related psychosocial hazards were contradictory, the results were more consistent in those studies that analyzed the differences between women and men in the exposure to long work hours, high physically demanding work, and noise, with more men than women exposed to these hazards. Conversely more women than men were found to be exposed to high job insecurity. Three of the 30 studies [48,49,51], showed that a higher proportion of women than men were exposed to high demand and low control, but two other studies [43,45] determined that there was a higher proportion of men who worked exposed to these conditions. None of these five studies had information about the statistical significance of differences observed in the prevalence. In addition, when high demand and low control were analysed separately, three studies [25,34,49] showed a higher proportion of women and four others [36,42,46,47] a higher proportion of men who worked exposed to high demand. However, most studies (six [34,36,42,46,47,49] out of seven) showed a higher proportion of women working exposed to low control and a higher proportion of men working exposed to low support (six [25,34,46-49] out of six). In addition, in two studies [36,37], one of which had a case–control design [37], a higher proportion of men than women were exposed to effort-reward imbalance. Three studies [43,48,51] found that a higher proportion of men than women were exposed to high physically demanding work and another three [42,44,49] found more men exposed to a high noise. However, no study was found showing a higher proportion of women than men exposed to these two hazards. Two studies [42,51] showed a larger proportion of women than men with exposure to high job insecurity.

### Employment conditions and gender inequalities in occupational health

Overall, employment conditions were less favourable among women. Two studies [41,46] showed a higher proportion of women than men working with no contract. However, none of them had available information about the statistical significance. While a higher proportion of women worked part-time (in three [32,46,51] out of three studies), or with a temporary contract (four [32,42,46,48] out of four studies) or a temporary fixed-term contract (one [41] out of one study); a higher proportion of men worked full-time (in two [32,46] out of two studies), or with a temporary non-fixed term (one [41] out of one) or permanent contract (two [32,41] out of two). In addition, a higher proportion of men than women worked as supervisors (two [45,51] out of two) or as upper managers (two [25,47] out of two).

### Work-related health problems and gender inequalities

Women had worse physical and mental health than men. Three studies [25,32,51] identified a higher prevalence of self-perceived poor physical health in women than men (only one of which was statistically significant) [25]; and five [32,40,41,46,54] found poorer self-perceived mental health in women than men (only one of which statistically significant) [40]. While seven studies [28,31,32,47,49,50,54] observed a higher proportion of women than men reporting any kind of musculoskeletal symptoms, five of which were statistically significant [28,31,49,50,54], no studies were found showing more men than women suffering these types of symptoms. A higher prevalence of poor mental health status in women than men was found in three studies [41,46,54], and a higher prevalence of mental health disorders [32,40], psychosomatic complaints [25,54] and self-reported occupational stress [30,51] was observed in two studies. No study found a higher prevalence in men than in women.

## Discussion

To our knowledge, this is the first study that attempts to identify the differences between women and men in the exposure to working and employment conditions through a systematic review of observational studies published in occupational health. This review, based on studies conducted mainly in Europe, shows that, as compared to men, women have greater feelings of high job insecurity, worse contractual working conditions and psychosocial work environment, and report poorer self-perceived physical and mental health. Conversely, as compared to women, men are exposed to longer work hours, high physically demanding work, noise, effort-reward imbalance and have higher job status. Both groups are exposed to high demands, but a higher proportion of women experience low control, and a higher proportion of men experience low support.

### Working conditions and gender inequalities

The majority of the results found on working and employment conditions could be explained by the powerful influence that employment, social class, and family exert on people’s everyday experiences, as well as the sexual-based division of labour that assigns different positions to men and women in these spheres of life. Moreover, these work-related gender inequalities in working and employment conditions are also linked to gender inequalities in power and resources. One of the most significant elements is the persistence of the ideology of domesticity, in which domestic work and childcare are normatively assigned to women [55].

The sexual-based division of work explains gender differences in time spent in paid work and other differences in working conditions. In this regard, the fact that we found a higher proportion of men than women who were exposed to a high level of noise at work could be explained by horizontal segregation. Horizontal segregation puts men in sectors of activity such as industrial environments and agriculture, as well as in mines, shipyards, and forges, where workers are exposed to this hazard to a much higher degree [11,12].

According to our findings, several studies have indicated that employed women experience worse psychosocial working conditions than employed men, and that a higher health burden might result from these exposures [19,20,56]. In addition, previous studies have found that men experience higher job demands, effort, and overcommitment; and lower social support at work; whereas women exhibit lower job control, higher emotional job demands and higher job reward [57-59]. In addition, women’s jobs are characterized by a greater level of monotony, with lower participation in planning, higher demands, more psychological and sexual harassment, higher exposure to the public, lower salaries, fewer prospects for promotion, and more precariousness than those of men [60]. The unequal gender distribution of work-related psychosocial hazards between women and men is mainly related to the horizontal segregation of the labour market, which concentrates women in occupations and economic activities (e.g. services) with higher exposure to work-related psychosocial hazards [6,61]. In addition, the unequal distribution of working tasks by gender within the same job title [11,13,14] may expose women to even higher levels of work-related psychosocial hazards [15]. Furthermore, vertical segregation, which places women in the lowest positions of the decision making scale, reinforces this effect [6]. It has been suggested that these inequalities put women at a higher risk of physical [62] and mental disorders [63], sickness absence [5], disability [64], and mortality [65] from work-related psychosocial hazards.

### Employment conditions and gender inequalities

Moreover, gender differences in power that place men in a better situation than women to bargain their employment conditions, could explain the gender inequalities identified in type of contract and job status, which show more men than women working with a permanent contract and occupying the higher job status positions. Vertical segregation and the “glass ceiling” phenomeno [6,7], a metaphor for the invisible barriers that prevent women from reaching positions of power that are occupied by men reinforce these two gender inequalities. These two gender inequalities could also be caused and maintained by the so-called “sticky floor” phenomenon [6], which prevents women from loosening the emotional ties that bind them to the rest of the members of the family unit. Furthermore, the modified male breadwinner model [55], in which males are engaged in paid work and work full-time, and their female partners are engaged in unpaid and paid work but work part-time, is very well represented in the results of our systematic review. These results in terms of the higher proportion of men working full-time and the higher proportion of women working part-time could also be partly explained by the gender division of social expectations among women and men in these two spheres of work and personal or family life [66]. In developing a flexible employment regime – which generally harms women more than men – employers could also exploit these differences in expectations towards work and employment [3,4]. This phenomenon could also explain why women perceive more job insecurity than men, and why men work longer hours in paid work than women. Women, more than men, may assume part-time work in an attempt to resolve their conflicts in balancing work and family life [55,67]. Although women’s conflicts in balancing work and family life could be improved by working part-time hours, part-time jobs are segregated into a narrower range of occupations than full-time jobs [67,68]. These narrow ranges of occupations are typically lower-paid, lower status, more monotonous, with fewer opportunities for advancement and related to job insecurity. In addition, part-timers have fewer social-work-benefits, less professional promotion, fewer opportunities to occupy managerial position in the company and are exposed to worse psychosocial work environments than full-timers [68-70].

Previous studies have found that more women than men work without a contract or with a temporary contract. This could be related to possible mechanisms of discrimination, which more women than men suffer in the workplace, specifically, the discrimination associated with gender [71,72]. This higher level of discrimination could be explained by horizontal and reinforced by the vertical segregation, which place women in job positions with less power compared to men. However, further analysis would be required to clarify this hypothesis.

### Work-related health problems and gender inequalities

Gender inequalities in health may result from the poorer working and living conditions of female workers. Although men experienced more physically demanding work than women, women experienced more musculoskeletal symptoms. This might be related to differences between women and men in the exposure to work-related hazards even working under the same job title [13,14]. For instance, the exposure to awkward working positions and repetitive movement with low loads is more common among women than men [67,73]. But, we have no information on these exposures in the studies included in our review. However, the gender differences related to the self-perceived physical health and musculoskeletal symptoms may also be influenced by a certain biological differences between women and men, which make women more susceptible than men to suffering musculoskeletal problems [74,75]. For example, there are anthropometric differences between men and women in muscle, fatty tissue and bone mass. Men have greater muscle mass and women have more fatty tissue [76]. In this regard, many workplaces and tools required by workers have been designed with men in mind, but without taking into account the anthropometric differences of women [50]. It is likely that both explanations – social and biological differences between women and men – complement each other. Moreover, the “double burden” phenomenon, which refers to the double exposure to the same occupational hazards at work and at home, such as housecleaning and caretaker tasks that mostly affect women [77,78], could explain these facts. For example, domestic work also implies exposure to ergonomic and psychosocial hazards, such as those related to informal care in families with disabled people that, besides physical and mental effort, often pose high emotional demands [67]. Women have a lower level than men not only of self-reported physical health, but also of self-reported mental health. This finding could perhaps be explained by the greater work-related psychosocial risk factors under which women work in the workplace and at home. Despite the dramatic increase of women in the labour market in recent decades, there have been no significant changes in the distribution of domestic work, even when both partners are working full time. Domestic tasks are still unequally distributed, with most of them (for example, caring for children, the elderly and disabled people) remaining women’s responsibility [67]. This fact reduces women’s recovery time after a day of work compared to men, a situation previous studies have associated with increased musculoskeletal symptoms [50].

Several previous studies show the influence of occupational social class on unequal gender distribution in the exposure to occupational hazards related to working and employment conditions [67,72,79-81]. For example, women in the most advantaged occupational social class – but not those in the most disadvantaged occupational class – are more likely to work in a worse psychosocial work environment than their male counterparts [72,81]. However, occupational social class as a determinant of gender inequalities in occupational social class only appear in our review in one study [42]. Thus, researchers should invest more efforts to incorporate the dimension of occupational social class in the analysis of gender inequalities in occupational health.

### Strengths and limitations

Some well-known gender inequalities such as discrimination [82], sexual harassment [83], bullying [84], and the gender-wage gap ratio in terms of median hourly wage for comparable work [85], which have also been cited as important work-related gender inequalities in occupational health in previous studies, were not identified in our review. This lack of findings could be due to either an artefact of the literature search strategy or the keywords used in our review, which was more focused on finding studies that analyze the different distribution among women and men in the exposure to occupational hazards in terms of working and employment conditions as determinants of gender inequalities in occupational health. Another explanation for this lack of findings could be that these aspects of discrimination, sexual harassment and bullying have been insufficiently investigated in the field of occupational health from a gender perspective, and more research is needed on these subjects. Although we found some studies in these fields, they were conducted only in women and thus were excluded from the review, since the gender perspective means that men must also be included in the analysis. Another limitation in this review is that some of the differences identified as existing between women and men in some studies were not statistically significant, or that a statistical analysis of significance was simply not conducted. However, the differences shown are present in the highest-quality studies available. Although MEDLINE and EMBASE include both biomedical and sociological references, the indexed scientific literature may not cover all investigations of the impact of gender inequalities as a determinant of occupational health. Therefore, many other studies that reflect gender inequalities may be also published in other documents that are less easily identifiable, the so-called grey literature. However, we applied a highly sensitive search strategy, which produced an optimal result in both databases. Another limitation could come from the possibility of missing articles published in languages other than English or Spanish. However, the search strategy was not initially limited by the language of the article, thus articles in a different language also followed the steps in the selection process. In addition, provision was made for translating and including any foreign-language article considered to be key in this field.

## Conclusions

Besides being a potential source of exposure to physical, hygiene, ergonomic and psychosocial hazards, work is one of the main axes that shapes life and identity, and its meaning differs by gender. Nowadays, in a context of transition from the traditional gender roles to more equal positions of men and women in society, employment has become more and more important in women’s lives, while family roles are expected to become more and more important to men. However, we have still identified a set of work-related gender inequalities in employment and working conditions and in reporting work-related health problems. Our findings are based on the scientific literature published on occupational health in the last decade. Knowledge of these work-related gender inequalities in health might be of use to researchers and practitioners in occupational health who wish to identify and monitor these factors, and for public policy makers whose goal is to attempt to reduce them.

## Competing interest

The authors declare that they have no competing interests.

## Authors’ contributions

CSJ, RPE and BFG participated in the design of the study. CSJ conducted the systematic search in the databases. CSJ and RPE assessed the quality of the articles. CSJ also identified gender differences in the prevalence of the main independent and dependent variables from the articles included in the review. CSJ, RPE, BFG, AL and MBE identified work-related gender inequalities and participated in discussions of the results. All authors have been involved in drafting and revising the manuscript. All of them have read and given final approval of the version to be published.
